# Netizens’ risk perception in new coronary pneumonia public health events: an analysis of spatiotemporal distribution and influencing factors

**DOI:** 10.1186/s12889-022-13852-z

**Published:** 2022-07-29

**Authors:** Yanling Li, Xiancong Wu, Jihong Wang

**Affiliations:** School of Public Administration and Law, Hunan Agricultural University, 1 Nongda Rd, Furong District, Changsha, 410128 Hunan China

**Keywords:** Risk perception, Internet search, Space-time distribution, Linear regression model, COVID-19

## Abstract

**Background:**

Internet search volume reflects the level of Internet users’ risk perception during public health events. The Internet search volume index model, an algorithm of concentration of Internet users, and statistical analysis of popular topics on Weibo are used to analyze the effects of time, space, and space-time interaction. We conducted in-depth research on the characteristics of the spatial and temporal distribution of Internet users’ risk perceptions of public health events and the associated influential factors.

**Methods:**

We analyzed the spatiotemporal distribution characteristics of Internet users’ risk perception after the Wuhan “city closing” order during the coronavirus disease 2019 (COVID-19) pandemic. We established five linear regression models according to different time periods and analyzed factors influencing Internet users’ risk perception by employing a Poisson and spatial distribution and topic modeling analysis.

**Results:**

Economy, education, health, and the degree of information disclosure affect Internet users’ risk perception significantly. Internet users’ risk perception conforms to the exponential distribution law in time and has periodic characteristics and stability trends. Additionally, Internet users’ average arrival rate dropped from week 1 to week 8 after the “city closing.” Internet users’ risk perception has a uniform distribution in space, economic and social development level distribution consistency, spatial agglomeration, and other characteristics. The results of the time-space interaction show that after 8 weeks of COVID-19, Internet search hot topics have become more stable, and Internet users’ information demand structure has become more rational.

**Conclusions:**

The Internet search cycle of the COVID-19 event is synchronized with the evolution cycle of the epidemic. The physical risk of Internet users is at the top of the risk structure, focusing on the strong concern about the government’s ability to control COVID-19 and its future trend. The government should strengthen network management; seize the risk control focus of key time nodes, regional locations, and information content of online communication; actively adjust the information content supply; effectively control the rebound of Internet users’ risk perception; establish a data-driven, risk-aware intelligence system for internet users; and guide people to actively face and overcome the potential risks and threats of COVID-19.

## Background

With the continuous development of big data and network technology, the media reports frequently on the occurrence of risk events. This may enhance people’s judgment on the probability of risk events and the severity of consequences, resulting in an excessive risk perception, triggering fear, helplessness, anxiety and other psychological reactions including mass panic, while a lack of information or asymmetric information transmission may make people’s risk perception low, resulting in negligence of risk prevention and control [1]. The information regarding “no evidence of human-to-human transmission” at the beginning of the coronavirus disease 2019 (COVID-19) outbreak in Wuhan, China led to a paralysis of the mind, and a golden opportunity to prevent and control the new coronary pneumonia (herein after referred to as COVID-19) was missed; the public health event outbreak that started in Wuhan in the early 2020s became prevalent in all municipalities and autonomous regions (hereafter referred to as provinces). In-depth research on the characteristics of spatial and temporal distribution of Internet users’ risk perceptions in public health events and their influencing factors is of great practical significance to further deepen the understanding of the COVID-19 event and how it influenced these perceptions. A better understanding of these characteristics can promote the government’s risk control of Internet users according to the law of spatial and temporal evolution. Therefore, this study analyzed the risk perceptions of Internet users during the COVID-19 event to identify factors that can improve risk control according to the law of spatial and temporal evolution. “Risk” in public health emergencies refers not only to the health risk to the public caused by the epidemic as a disease but also to the potential loss to the public in the areas of production, life, and consumption.

Severe acute respiratory syndrome coronavirus 2 (SARS-CoV-2), the causative agent of COVID-19, is novel coronavirus that was not previously detected in humans. On January 30, 2020, the World Health Organization declared COVID-19 as a “Public Health Emergency of International Concern [2].” Confirmed COVID-19 cases have been found in at least 130 countries and regions worldwide. In China, confirmed cases were reported in 31 provinces (including municipalities and autonomous regions) across the country. As of March 22, 2020, at midnight, a total of 687,680 COVID-19 close contacts, 81,054 confirmed COVID-19 cases, and 3261 cumulative deaths owing to COVID-19 were reported nationwide [3]. After the COVID-19 outbreak, a large number of studies were conducted worldwide. By the end of February 2020, more than 100 related academic papers were published in authoritative journals including *Nature*, *Science*, *Cell*, *The Lancet*, *New England Journal of Medicine*, and *Chinese Public Health* as well as in *BioRxiv* and *MedRxiv* preprint platforms. These papers focused on key issues, such as pathogen identification, virus traceability, transmission mechanism and prediction models, drug treatment, and vaccine development. Among these, the most debatable was the prediction of the virus transmission trend. Chinese infectious disease experts Zhong Nanshan and Li Lanjuan calculated that the basic reproduction number of the virus is between 2 and 3 (i.e., one infected person can infect 2–3 people), determined that the incubation period of COVID-19 infection in humans is generally 14 days, and predicted that the peak period of infection would be in mid-February 2020 [4]. These results indicate that under the “Chinese” prevention and control model, the transmission peak would be approximately 8 weeks after the start of the COVID-19 outbreak in mid-December 2019. In another study, the China Center for Disease Control and Prevention predicted an R_0_ value of 2.2 and an incubation period of approximately 14 days for COVID-19 infection in humans [5]. In a report published in *The Lancet* by the team of Dean Gabriel Leung of the Li Ka Shing Faculty of Medicine, The University of Hong Kong [5], the results of a predictive model constructed by the World Health Organization Centre for Infectious Disease Modelling, in collaboration with the Medical Research Council Centre for Global Infectious Disease Analysis at Imperial College, UK [6] concluded that the incubation period for COVID-19 infection in humans is approximately 14 days (2 weeks).

After the COVID-19 outbreak, China adopted strong community-based home quarantine measures and achieved effective disease prevention and control. Many scholars have showed concern about the Chinese model of “community-based home quarantine,” including Wuhan’s city closing(CC) control and the national “containment and grounding” method [7] Hu Yang proposed that the core purpose of “city closing” was to close the exit routes from the infected areas to cut off the nationwide spread of the source of the viral infection [8]. Ruan Shigui proposed that when a new infectious disease breaks out in one place, it spreads from this source of infection like in epidemic wave to other places as the population migrates [9], and Pyle believed that one way of controlling the spread of an epidemic wave is adopting different control measures [10]. Other scholars have also attempted a phased approach to determine the number of confirmed COVID-19 cases based on equations derived from general models, and the results predicted that COVID-19 prevention and control would be better managed approximately 8 weeks after the outbreak under the Chinese “community-based home quarantine” model [11]. Some scholars have investigated the Chinese public perception of the disease and the public response during the COVID-19 outbreak, analyzed the influence of online public opinion on the prevention and control of COVID-19, and obtained optimistic results on the ability and effectiveness of online public opinion management by the Chinese government [12].

### Theoretical foundation

Risk perception is an important concept in risk research. Since risk perception comes from peoples’ subjective judgment, there is often a gap between perceptions and objective real risk [13]. The risk perception of Internet users in public risk events shows the basic characteristics of two-dimensional influence in time and space. The internal and external factors affecting the risk perception of Internet users are very complex. These factors include the evolution of public risk events themselves, a lack of information, the spread of Internet rumors [14], the physiological and psychological reactions of Internet users in the process of perceiving risk, and the combined influence and interaction of social factors such as media, culture, economy and politics, as well as the socio-economic background factors of Internet users themselves [15].

The temporal distribution of risk perception has an obvious regularity. Risk perception involves the process of collecting, selecting, and judging signals about the impact of the uncertainty of risk events, and these signals include both direct and indirect experience. The factors affecting risk perception can be divided into the following categories: risk factors, information factors, personal factors, and environmental factors, among which information factors are related to indirect experience, and individuals without direct experience build their understanding of risk on external information [16]. In this study, we used sequence data from information searching in the Internet environment to focus on the time-evolving characteristics of the risk perception of Internet users concerning public health events.

The spatial effect of risk perception is important. As a public health event evolves, there is a shift in concentration of groups of Internet users who perceive risk and communicate this. Drawing on John Friedmann’s core periphery theory, it can be found that the most effective communicators on a media networks tend to be located within the core of the network. When multiple effective communicators exist at the same time, the distance between them becomes a key parameter in determining the success or failure of diffusion [17]. Risk perception is usually associated with uncertainty or adverse consequences. If an individual’s cognitive state is unable to make judgments about the incoming information (i.e., is in a situation of apparent perceived risk), the individual eliminates his or her cognitive uncertainty by obtaining relevant information from the outside (i.e., by migrating and adopting information in the community of agents of online media platforms for an analytical opinion) [18]. Since one of the ways to reduce risk perception is to obtain more information, risk perception can increase information searching [19]. Therefore, the spatial effect of risk perception of Internet users during the evolution of public risk events can be reflected by the concentration of Internet user groups, that is, the density of Internet searches in different communities calculated after filtering out the effect of community population size on the volume of Internet searches.

Based on the two dimensions of time and space, risk perception also has a space-time interaction. In the context of big data, people are confronted with two “risk fields” that are clearly differentiated and completely different in nature, but intertwined and coexisting, namely, the “offline” real space and the “online” virtual space on the Internet. Using social network analysis, it is found that in a large number of real complex networks, a very small number of nodes have a very large number of connections, while the majority of nodes have only a few connections, and the degree of distribution of network nodes is power-law distributed. In addition, the nodes also show the phenomenon of aggregation, forming so-called “social circles” or network communities on the network. The formation of such groups or communities has both a temporal extension and a spatial migration effect [20]. In the online media platform, space-time interactions between groups lead to the emergence of hierarchical structures in social networks due to the fractal nature of this self-similarity [21]. However, the literature on space-time interactions of online risk perception is not extensive, and a research method characterized by “topic models” has been more often used to examine the space-time interactions of online “social circles” over time.

## Methods

### Analysis framework

#### Analysis of temporal distribution

The Poisson distribution is used to describe the probability of an event occurring specifically during a certain period of time. Generally speaking, the risk perception of Internet users in a risky event conforms to the homogeneous Poisson process in temporal distribution [1]. Assuming that Internet users perceived the risk of the event on the day of the “CC” order in Wuhan during the COVID-19 outbreak, we define the probability of their Internet search at time t to t + Δt as λ. Here we set Δt to be 7 days, or 1 week. Then, the time at which the Internet search occurs can be viewed as an exponential distribution process. Let the probability density function of the exponential distribution be:





where t > 0 and λ > 0. The evolution of Internet users’ risk perception of COVID-19 over time is reflected in the value of λ: the larger the search volume, the faster the rate of decline, and the higher the risk perception. λ is the average arrival rate of Internet users, and the larger the value of λ, the faster the risk perception of Internet users is likely to improve after the COVID-19 outbreak. In other words, the greater the probability of Internet users conducting an Internet search over time, the higher the degree of Internet information disclosure; that is, the greater the density of the Internet search, the more transparent the information, and the stronger the Internet users’ risk perception.

#### Analysis of spatial distribution

The spatial distribution of Internet users’ risk perception refers to the status of spatial migration of the Internet users’ groups most concerned about the evolution of events, as marked by the intensity of online information search volume. In this paper, we extended the algorithm of Arunima et al. on the concentration of Internet users’ groups [22]. First, we obtained the search data of COVID-19 information by Internet users in each province of China in general, and after filtering out the influence of the community population on the Internet search volume, we calculated the Internet search volume density of Internet users nationwide and in different provinces. Finally, we ranked the Internet users in different provinces according to their Internet search volume density and observed the spatial migration status of Internet users in provinces with a relatively higher ranking of search volume density. The spatial plane distribution structure of Internet users’ perception of COVID-19 risk was finally derived. The network search volume algorithm is as follows, first calculating the information demand of Internet users:

$${\mathrm{I}}_{\mathrm{i}}={\mathrm{search}}_{\mathrm{i}}/{\mathrm{online}}_{\mathrm{i}}$$

where Ii is the information demand of Internet users in province i, searchi is the Internet search volume of COVID-19 by Internet users in province i, and onlinei is the number of Internet users in province i. Since the age structure of the population in each province is very close and the proportion of China’s Internet users to the total population is also high, this research approximates the number of Internet users by the number of people aged 15–64 in province i (the same below). In order to reflect the relative size of the COVID-19 risk perception of Internet users in each province, we then calculated the proportion of COVID-19 information demand of Internet users in each province of the total information demand of Internet users nationwide, which was calculated by the following formula:

$${\mathrm{RI}}_{\mathrm{i}}=\left({\mathrm{I}}_{\mathrm{i}}/\sum {\mathrm{I}}_{\mathrm{i}}\right)\ast 100\%$$

where we defined RI_i_ as the density of Internet search for COVID-19 information by Internet users in province i, Ii as the COVID-19 information demand of Internet users in province i, and ∑I_i_ as the total information demand of Internet users nationwide.

Finally, we further analyzed the distribution structure of the risk perception of COVID-19 in the spatial plane by ranking the COVID-19 network search volume density (RI_i_) of the Internet users in each province and further analyzed the distribution structure of the risk perception of COVID-19 in the spatial plane of the Internet users in different provinces according to the RI_i_ ranking results, that is, the distribution of the maximum and minimum RI_i_. Of course, with the evolution of COVID-19 events, this spatial plane distribution structure will be displaced over time. To further observe the connection between the changes in the spatial and temporal distribution of risk perceptions of Internet users, we need to observe the changing status of offline events related to the COVID-19 pandemic at the same time. We analyzed the space-time evolution characteristics of Internet users’ risk perceptions through a comparative study of offline and online events related to the COVID-19 pandemic.

#### Analysis of space-time interaction

The space-time interaction of Internet users’ risk perceptions in public risk events is very complex to research. In recent years, “Topic Models” have been applied to such studies. The theory of “topic models” suggests that the space-time migration of social network users’ interests is potentially related to the process of topic dynamics [23]. The “topics” on the Internet are often a concentrated expression of the evolution of Internet users’ risk perceptions in time and space [24]. Influenced by the realistic political, economic, social, and even cultural differences in different regions, we accepted the Topic Models notion that “topics” focus on the spatial and temporal shifts of Internet users’ interests, and therefore, we conducted a statistical analysis of “topic” content on the interaction of “time” and “space” dimensions of Internet users’ risk perceptions during the COVID-19 event. Instead of using a more in-depth “topic model,” this research only classifies and labels the content of Internet users’ topics in the COVID-19 event and uses a simple statistical analysis method of hot topics to analyze the spatial and temporal interactions of Internet users’ risk perceptions in the event. Since the risk perceptions of Internet users in public risk events are interdependent and combined with each other in two basic dimensions of “time” and “space,” they form the basic space-time structure of the distribution of Internet users’ risk perceptions, that is, the so-called risk perception environment of Internet users. The differences in the risk perception environment of Internet users may interfere with the space-time interaction function of risk perception, thus forming different space-time structures. Therefore, this research selected the information demand for the “topic” of the COVID-19 event as a proxy variable and established the analysis logic of “topic demand - drive environment change - changed environment leads to new topic demand - then drive new environment change.” By observing the transfer of information on COVID-19 “topics,” we analyzed the results of space-time interaction of Internet users’ risk perception.

### Variables & samples

#### Variable definitions and relationship hypotheses

The risk perception of a public health event is composed of the objective risk brought about by the event itself and the public’s subjective perception about the risk of the event, with the former deriving from objective facts and the latter being influenced by personal factors, social and economic factors, and so on [25]. Integrating the existing studies, this research selected four individual variables as influencing factors of Internet users’ risk perceptions in public risk events: (1) gender ratio of men and women, (2) age structure, (3) years of education per capita, and (4) mortality rate reflecting regional health level. The definition and relationship assumption of each variable is shown in Table 1.

**Table 1:** Variable definitions and relationship hypotheses

Variable types and names	Definition of variables (quantitative units or symbols)	Relationship Hypotheses^a^
Explained variables:		
Internet search volume density *Y*_*i*_ (i.e., *RI*_*i*_)	The proportion of information demand of Internet users in each province to the total information demand of Internet users nationwide (%)	
Explanatory variables:		
Male to female sex ratio (*X*_*1*_)	Male share of total population (%)	Higher risk perception among men (+)
Age structure (*X*_*2*_)	Population aged 15–64 as a percentage of total population (%)^b^	The higher the ratio, the higher the risk perception (+)
Years of education per capita (*X*_*3*_)	Years of formal education only (Years)	The longer the number of years of education, the higher the risk perception (+)
Mortality (*X*_*4*_)	Ratio of the number of deaths by province in a year to the average number for the same period (%)	The higher the mortality rate (lower the regional health level), the lower the risk perception (−)
Per capita GDP (*X*_*5*_)	Final results of production activities of all resident units in the provinces during the year (billion yuan, RMB)	Higher risk perception in provinces with higher GDP (economically developed areas)
Rate of decline in risk perception (*X*_*6*_) (*λ* value)	The probability of Internet users searching for the COVID-19 event reflects the extent of Internet information disclosure	The higher the search volume, i.e., the faster the rate of decline, the higher the risk perception (+)

^a^In the relationship hypotheses, “+” indicates the explanatory variables have a positive correlation with the explained variables, and “-” indicates a negative correlation

^b^This paper defines Internet users aged 15–64 as Internet users with sanity. According to this definition, the search volume of Internet users collected in this paper may have a small systemic error

Table 1 presents six hypotheses based on available studies: first, men have a higher disaster reduction knowledge base than women [26]. Second, the higher the proportion of the population aged 15–64 years in the age structure of the population, the higher the risk perception. Third, the higher the number of years of education (school age), the higher the risk perception. Fourth, the higher the mortality rate, the lower the regional health level, and the lower the risk perception. Fifth, the higher the gross domestic product (GDP) of the province (economically developed areas), the higher the risk perception [27]. Sixth, the faster the rate of decline in risk perception, the higher the risk perception [28].

#### Sample selection

We studied the spatial and temporal distribution of Internet users’ risk perception of the COVID-19 event, taking the evolution of the weeks after the CC order as the time scale and the provinces of China as the regional spatial scale. All of the methods used were in accordance with the Declaration of Helsinki. The weekly search density and the number of new confirmed cases of COVID-19 events in 31 provinces in China within 8 weeks were selected as samples to analyze the spatial and temporal evolution of risk perception of Internet users after the occurrence of COVID-19 public health events, including the spatial and temporal evolution characteristics and influencing factors as well as their effects.

### Empirical analysis model

Relevant studies generally reflect that the evolution of Internet users’ risk perception of public health events is characterized by a high degree of coincidence with the evolution of risk sources, that is, the synchronization of online and offline evolution [13]. Data published by the National Health Commission, PRC, shows that since March 19, 2020, there have been zero new confirmed cases in mainland China (with only a few confirmed cases imported from abroad), also reflecting that the peak of COVID-19 transmission in mainland China was at the two-month (8 week) time point after the outbreak, with an inflection point trend in all infectious indicators during the fifth week. Based on these empirical research results and disease transmission data, this research draws on the perceived risk model of Dowling et al. [29], using 1 week, 2 weeks, 5 weeks, and 8 weeks after the COVID-19 outbreak as respective time windows. We also used the collected and calculated cross-sectional data of X1, X2, X3, X4, X5 (from the *China Statistical Yearbook*), and X6 (calculated by MATLAB software) in 2018, using Eviews8.0 software, respectively, to build the five linear regression models according to different time periods of the factors influencing the risk perception of Internet users in each province of China during the COVID-19 event.

### Data description and statistical results

#### Data source

We used the Baidu search engine to obtain COVID-19 Internet search density data on January 23, 2020, the day of the CC order, and for the following 8 weeks. The daily data of the number of new confirmed cases in each province of China published by the National Health Commission, PRC, and the latest data of X1, X2, X3, X4, X5 for 31 provinces of China in 2018 from the *China Statistical Yearbook*, and X6 data were calculated by MATLAB software. Baidu Index is a data-sharing platform based on Baidu’s massive internet users’ behavioral data. The main function modules include, but are not limited to, trend research based on individual words (including overall trends, personal computer trends, and mobile trends), demand mapping, public opinion manager, crowd portrait, etc.

**Fig. 1:**
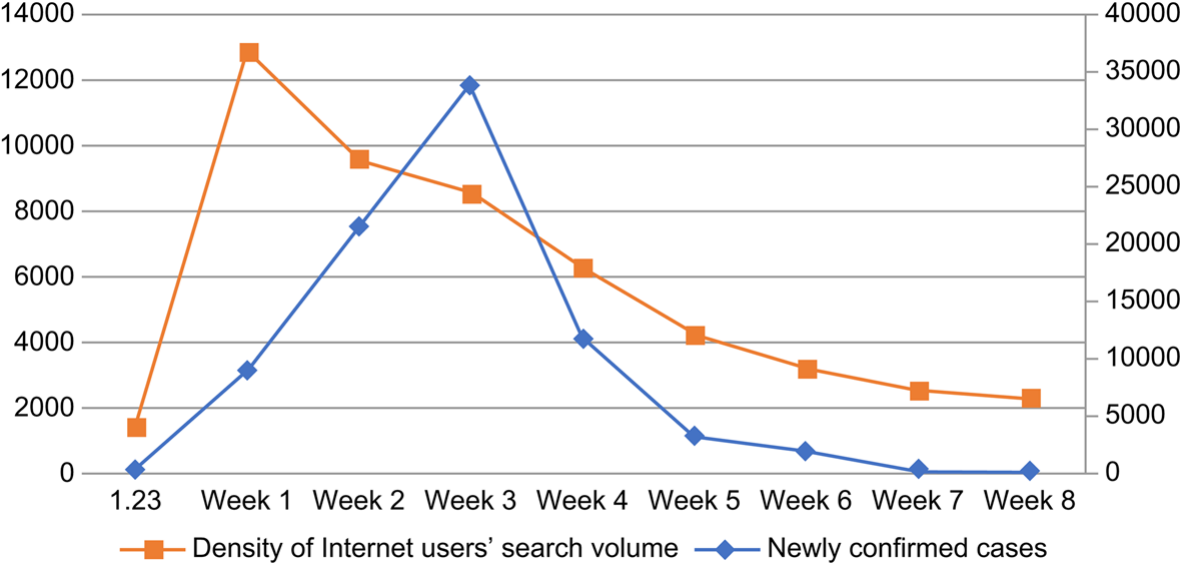
Trends of internet users’ perception and epidemic changes during the COVID-19 pandemic. Trends in risk perception and offline epidemic among netizens in the 8 weeks after the COVID-19 event in Wuhan. The time duration in the above figure is within 8 weeks after the “city closing” in Wuhan

**Fig. 2:**
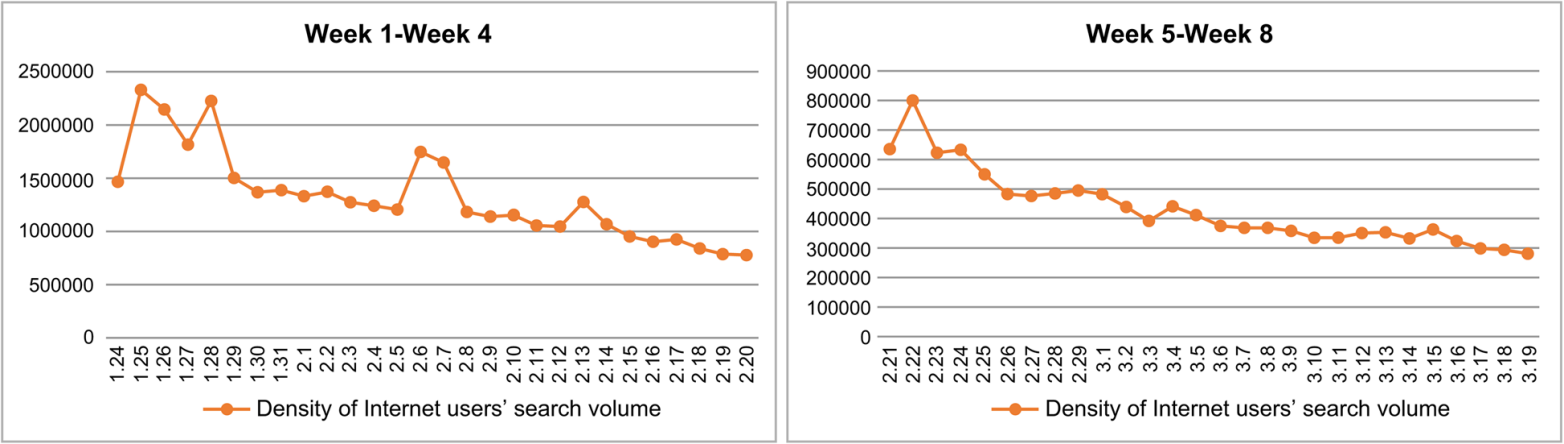
Trend in daily risk perception of netizens after the COVID-19 event in Wuhan. The time duration shown is only 8 weeks (56 days) to emphasize the daily change in the density of Internet users’ search after the “city closing” in Wuhan

The weighted sum of the search frequency of each keyword in the Baidu web search was analyzed and calculated using MATLAB software with the keyword, “COVID-19” as the statistical object. In order to observe the search volume of Internet users within 8 weeks after the CC order, we plotted the risk perception of COVID-19 netizens and its trend with the offline epidemic during the 8 weeks from January 24 to March 19, 2020 after the CC order (see Fig. 1). For a more detailed observation, we segmented the time by day to obtain Fig. 2, which can be used to better conduct an analysis of the trend of Internet users’ online Internet search volume after the COVID-19 outbreak by day. To further analyze the space-time interaction of Internet users’ risk perceptions, we collected online Internet microblog topic heat data after the COVID-19 outbreak based on Weibo. Microblog topic heat included the number of reads and the number of discussions. The number of reads is the sum of the total number of times the topic was read in each scenario within the microblogging platform. The number of discussions is the total amount of microblogs with topics involved in discussions; microblogs include original microblogs and retweeted microblogs. We then collected 349 highly relevant microblog topics related to COVID-19 from Weibo, with keywords such as new crown, pneumonia, virus, epidemic, medical care, Hubei, and Wuhan, among others, which were read more than 100 million times and discussed more than 10,000 times between January 9 and March 19, 2020. We sorted these microblog topics according to the sum of the number of reads and discussions that decided how hot they were.

### Data description

#### Description of the temporal evolution of internet users’ risk perceptions

The trends of risk perception of COVID-19 netizens and the changes in the offline epidemic during the 8 weeks from January 24 to March 19, 2020, after the CC order are shown in Fig. 1 below. Furthermore, the trend of the change of Internet users’ search volume density by day segment following the issuance of the CC order of Wuhan is shown in Fig. 2 below. In particular, for aesthetics and clarity, Fig. 2 shows the density of Internet users’ search volume for 8 weeks (56 days) in two line graphs, each of which spans 4 weeks (28 days).

#### Description of the spatial distribution of internet users’ risk perception

Based on the search volume of Internet users on Baidu’s webpage, the descriptive statistical results of RIi values of the density of Internet search volume for the risk perception of COVID-19 Internet users in each province were calculated (see Table 2). The web search volume density of Internet users reflects the proportion of Internet users with COVID-19 information searches in each province to the total number of Internet users in that province after filtering out the influence of population size on search volume. Table 2 reports the descriptive statistical results for weeks 1, 2, 5, and 8 after the CC order.

**Table 2:** Descriptive statistics of internet search density on COVID-19 events after “CC” in Wuhan

Observation time	Internet users network search volume density/province name		Descriptive statistics results		
	Max	Min	Mean	Range	Standard deviation
Total national two-month period (8 weeks in total)					
Internet search volume density (*RIi*)	Beijing	Xinjiang	5.2	8.83	1.71
	7.84%	1.98%			
Number of new confirmed cases (persons)	Guangdong	Qinghai	431.62	1278	392.76
	1295	17			
The day after the outbreak (Jan 23^rd^)					
Internet search volume density (*RIi*)	Beijing	Guangdong	0.1366	0.058	0.34
	8.85%	0.33%			
Number of new confirmed cases (persons)	Chongqing	Shanxi, Zhejiang, Shandong, Guangdong, Guizhou, Yunnan, Shaanxi, Gansu, Qinghai, Xinjiang	3.65	18	4.85
	18	0			
First week after the outbreak					
Internet search volume density (*RIi*)	Beijing	Xinjiang	1.31	2.11	0.38
	7.41%	1.86%			
Number of new confirmed cases (persons)	Zhejiang	Qinghai	119.76	487	120.45
	494	7			
Week 2 after the outbreak					
Internet search volume density (*RIi*)	Beijing	Xinjiang	1	1.75	0.31
	8.04%	2.02%			
Number of new confirmed cases (persons)	Guangdong	Qinghai	176.07	615	174.52
	625	10			
Week 5 after the outbreak					
Internet search volume density (*RIi*)	Beijing	Guangxi	0.43	0.78	0.16
	8.44%	2.21%			
Number of new confirmed cases (persons)	Guangdong	Inner Mongolia, Liaoning, Jiangsu, Hainan, Guizhou, Yunnan, Shaanxi, Gansu, Qinghai, Xinjiang	3.52	15	4.61
	15	0			
Week 8 after the outbreak					
Internet search volume density (*RIi*)	Beijing	Xinjiang	0.23	0.51	0.11
	9.34%	1.83%			
Number of new confirmed cases (persons)	Beijing	Hebei, Shanxi, Inner Mongolia, Liaoning, Jilin, Jiangsu, Anhui, Fujian, Jiangxi, Henan, Hunan, Hainan, Chongqing, Guizhou, Qinghai, Ningxia, Xinjiang	4.69	49	11.76
	49	0			

The data of the first week after the “city closing” of Wuhan is the average data of seven days in that week, and so on; The number of new confirmed cases in a week is the sum of the number of new confirmed cases per day in that week; The mean, range, and standard deviation in the descriptive statistical results refer to the statistical results of the national provinces with provinces as samples; We excluded the data of Hubei Province and Tibet Autonomous Region and did not include them in the comparison of provinces throughout the country

According to the incubation period characteristics of the COVID-19 outbreak, we classified the RIi values of Wuhan on the day after the CC and in the first, second, fifth, and eighth weeks after the CC into four intervals: RIi≧7.5%, 7.5 > RIi ≧5, 5 > RIi≧2.5, 2.5 > RIi > 0. The corresponding risk perception levels of Internet users are defined as I, II, III, IV, and so on. The statistical results of the RIi value partition are shown in Table 3.

**Table 3:** Calculation results of partition statistics of RIi values

Jan 23		Week 1		Week 2		Week 5		Week 8	
*RIi* value	New confirmed cases (persons)	Weekly *RIi* mean	New confirmed cases (persons)	Weekly *RIi* mean	New confirmed cases (persons)	Weekly *RIi* mean	New confirmed cases (persons)	Weekly *RIi* mean	New confirmed cases (persons)
> = 7.5% Risk Perception Level I									
Beijing	12			Beijing	160	Beijing	14	Beijing	49
5% ~ 7.5% Risk Perception Level II									
Shanghai	4	Beijing	103			Shanghai	3	Shanghai	25
								Zhejiang	7
2.5% ~ 5% Risk Perception Level III									
Zhejiang	0	Zhejiang	494	Shanghai	141	Zhejiang	2	Guangdong	37
Hainan	4	Shandong	154	Zhejiang	469	Guangdong	15	Tianjin	1
Jiangsu	8	Liaoning	41	Tianjin	48	Chongqing	9	Hainan	0
Chongqing	18	Shanghai	108	Shandong	201	Jiangsu	0	Jiangsu	0
Shandong	0	Jiangsu	159	Liaoning	39	Tianjin	5	Chongqing	0
Fujian	3	Hebei	80	Hebei	89	Shandong	8	Fujian	0
Shaanxi	0	Chongqing	179	Jiangsu	240	Sichuan	13	Qinghai	0
Sichuan	10	Tianjin	26	Jilin	51	Hebei	5	Liaoning	0
Anhui	6	Anhui	222	Chongqing	205	Hainan	0	Shandong	2
Hebei	1	Hainan	41	Ningxia	22	Fujian	3	Sichuan	2
Tianjin	1	Sichuan	163	Qinghai	10	Liaoning	0	Hebei	0
Jiangxi	4	Jiangxi	233	Sichuan	167	Jilin	2	Ningxia	0
Liaoning	2	Qinghai	7	Hainan	62	Ningxia	1	Jilin	0
Hunan	15	Jilin	11	Anhui	428	Qinghai	0	Guizhou	0
Shanxi	0	Fujian	114	InnerMongolia	28	Anhui	2	Shaanxi	1
Henan	4	InnerMongoli	14	Guangdong	625	InnerMongolia	0	Jiangxi	0
Qinghai	0	Guangdong	295	Jiangxi	423	Gansu	0	Heilongjiang	2
Heilongjiang	2	Shanxi	38	Guizhou	62	Shanxi	1	InnerMongolia	0
Guizhou	0	Ningxia	19	Shanxi	57	Guizhou	0	Shanxi	0
Gansu	0	Shaanxi	60	Shaanxi	111	Jiangxi	1		
InnerMongolia	1	Hunan	308	Fujian	104	Henan	5		
Jilin	2	Heilongjiang	55	Heilongjiang	218	Hunan	6		
		Guizhou	12	Henan	513	Heilongjiang	1		
		Gansu	27	Hunan	440	Shaanxi	0		
				Gansu	33				
0 ~ 2.5% Risk perception level IV									
Xinjiang	0	Henan	343	Yunnan	53	Xinjiang	0	Henan	0
Ningxia	1	Yunnan	78	Guangxi	85	Yunnan	0	Hunan	0
Guangxi	8	Guangxi	74	Xinjiang	22	Guangxi	0	Guangxi	1
Yunnan	0	Xinjiang	15					Gansu	7
Guangdong	0							Yunnan	2
								Anhui	0
								Xinjiang	0

RIi values are listed in order of magnitude by province within the same interval. The weekly RIi mean value refers to the weighted average of that week. New confirmed cases refer to the cumulative number of cases in that week

#### Space-time description of the shift of “topic heat” in internet users’ risk perception

The space-time interaction of the risk perception of Internet users was analyzed based on the logic of “topic demand-drive environment change-changed environment leads to new topic demand-drive new environment change” by observing the “topic heat” shift of COVID-19 information. A microblog topic (hereinafter referred to as topic) is a topic page related to a certain topic based on the content of various channels such as microblog hotspots, personal interests, and netizens’ discussions, which is modified and edited by topic moderators. Microblog users can enter this page to post tweets for discussion, and the topic page will also automatically include related tweets containing the topic term.

We collected 349 highly relevant microblog hot topics about COVID-19 from Weibo, with keywords such as, new crown, pneumonia, virus, epidemic, medical care, Hubei, Wuhan, and others, which were read more than 100 million times and discussed more than 10,000 times between January 9 and March 19, 2020. We only compared 28 of them, which had a total number of public opinions (the sum of the number of reads and discussions) exceeding 500 million, accounting for 8% of the total number of hot topics. We ranked the hot topics of these microblogs in descending order according to the total number of public opinions, then used Python Baidu software crawler to crawl the information of netizens’ risk perception of COVID-19 for emotional polarity classification. Each hot “topic” was labeled and subdivided, and the three types of information demanded by netizens about the latest news of COVID-19 (mainly referring to the domestic infection situation and the effect of government control), COVID-19 evolution prediction and prevention and control (mainly referring to expert judgment), and COVID-19 transmission direction (mainly referring to the transmission and import situation out of China) were labeled as categories 1, 2, and 3, respectively. We used the Baidu index search function to search these three types of keywords to obtain the statistical results of COVID-19 event hot topics in each province within 8 weeks as shown in Table 4 the Baidu Index is a data sharing platform based on Baidu’s massive Internet users’ behavior data, and its main function modules include, but are not limited to, trend research based on individual words (including overall trend, personal computer trend, and mobile trend), demand mapping, public opinion manager, crowd portrait, and so on. Among these, there were 15 topics in category 1 (53.6%), 8 topics in category 2 (28.6%), and 5 topics in category 3 (17.9%). In terms of time segmentation, among the 28 hot “topics,” there were 3 in the 1st, 3rd, 6th, and 7th weeks, 6 in the 2nd week, 4 in the 4th week, only 1 in the 5th week, and 5 in the 8th week.

**Table 4:** Topics of COVID-19 event that netizens were concerned about after the “city closing” in Wuhan

Topics	Week	Public opinion ranking	Total public opinions (million times)	Public opinion categories	Topics	Week	Public opinion ranking	Total public opinions (million times)	Public opinion categories	
Shuanghuanglian inhibits 2019-nCoV	2	1	2220.555	1	CCTV reporter visited the Wuhan Red Cross	2	5	850.335	1	
Dr. Li Wenliang dies	3	1	2011.394	1	Large number of 2019-nCoV present in South China seafood market	1	3	830.144	1	
Epidemiologist says epidemic cannot wait	1	1	1640.293	2	Zhang Wenhong predicts eventual development of new coronavirus	6	2	820.116	2	
The epidemic is still spreading	1	2	1590.255	1	Experts advise teachers to wear masks after school starts	5	1	810.076	2	
Many places clarify school start time	8	1	1550.123	1	Zhang Wenhong said the epidemic is basically impossible to end this summer	8	2	800.085	2	
Hubei deputy governor responds to Wuhan residents’ online requests for help	2	2	1440.396	1	Children of front-line volunteers in Hubei added 10 points for admission to the high school entrance examination	4	4	780.079	1	
New pneumonia help channel opens	2	3	1304.721	1	Zhong Nanshan talks about specific antiviral drugs	2	6	770.06	2	
Wuhan will be held accountable for finding home-diagnosed patients	4	1	1180.052	1	Trump declares national emergency in response to epidemic	8	3	750.051	3	
16 provinces a province package a city to support Hubei	3	2	1120.192	1	Trump praises China’s epidemic prevention and control achievements	8	6	680.061	3	
Zhong Nanshan talks about the peak of the new crown pneumonia epidemic	4	2	1020.082	2	South Korea confirms for the first time that Xintiandi believers have been to Wuhan	6	7	680.023	3	
Change in responsibilities of main comrade of Hubei provincial party	3	3	970.113	1	Zhong Nanshan says end of epidemic is to be expected in June	7	3	590.024	2	
Italian residents refuse to wear masks	6	1	960.099	3	Italy’s mask supply goes missing after being withheld by Germany	8	10	570.018	3	
The first person to report the epidemic is lauded by Hubei Provincial Department of Human Resources and Social Security and Hubei Provincial Health Commission.	2	4	870.08	1	Zhong Nanshan says foreign epidemic much like early Wuhan situation	7	6	546.014	2	
Autopsy of the body of the first patient who died from COVID-19 in China	4	3	860.077	1	Congressional physicians predict nearly half of Americans could be infected	7	9	520.014	3	

Only topics with total public opinion of 500 million or more are counted

### Goodness of fit of internet search volume index model for internet users’ risk perception

Based on the risk perception data of COVID-19 captured by MATLAB software in Fig. 1, an exponential distribution model reflected by λ values over time was constructed. The index model was fitted to the weekly Internet search data of the day, the 1st week, the 2nd week, the 5th week, and the 8th week after the CC order was issued, and the fitting results are shown in Table 5.

**Table 5:** Simulated goodness of Internet users’ risk perception index of COVID-19 city closing in Wuhan

	Wuhan “city closing” day	Week 1	Week 2	Week 5	Week 8
λ	0.153	0.337	0.315	0.169	0.034
Adjusted R^2^	0.831	0.875	0.903	0.914	0.922

### Linear regression analysis of factors influencing internet users’ risk perceptions

Eviews8.0 software was used to analyze the correlation between Internet users’ risk perceptions and relevant variables in each province of China. Eq. I, II, III, IV, and V of the linear regression model of the factors influencing Internet users’ risk perceptions in the COVID-19 event were demonstrated. In each province of China, the equations were established using the day of the COVID-19 outbreak (January 23), 1 week, 2 weeks, 5 weeks, and 8 weeks after the outbreak as the time windows; the results of the model regression analysis are shown in Table 6.

**Table 6:** Linear Regression Model Analysis of Internet Users’ Risk Perceptions in the COVID-19 Event

Variable	Equation I	Equation II	Equation III	Equation IV	Equation V
X_1_	0.00135 (0.613959)	3.77E-02^a^ (1.991287)	0.006534 (0.83375)	1.71E-03 (1.64111)	0.001736 (1.86153)
X_2_	0.060085 (1.486521)	0.001965 (1.825641)	7.66E-04 (1.852471)	1.003657^a^ (2.663358)	0.904527 (1.375526)
X_3_	8.257481^b^ (2.307547)	1.10E-06^a^ (2.085241)	-0.82E-03 (−1.754117)	0.066856^a^ (2.15417)	1.021367^a^ (2.875502)
X_4_	-1.214574^a^ (−1.985114)	-1.70063^b^ (−2.425471)	-1.004772^a^ (−2.036574)	−0.001845 (−1.00036)	4.54E-05^b^ (2.000067)
X_5_	6.85E-02^b^ (4.195523)	3.345E-02^c^ (5.145672)	2.87E-05^c^ (4.837169)	1.66E-04^b^ (2.105878)	1.37E-05^a^ (2.052741)
X_6_	1.978421^b^ (2.854632)	1.003527^c^ (3.745811)	1.643589^b^ (2.107952)	0.000275 (1.795422)	1.087235^a^ (2.111253)
Adjusted -squared	0.685411	0.073251	0.453375	0.268742	0.204685
F-statistic	10.852311^c^	3.114785^b^	2.728768^a^	5.416758^c^	3.000256^b^

^a, b, c^ in the table indicate statistically significant at the 10, 5, and 1% levels, respectively

## Results

### Linear regression analysis of factors influencing internet users’ risk perceptions

Table 5 shows that the Internet search volume data of the five-time windows of the COVID-19 event after the CC order had a high fit to the index model, and the risk perception of Internet users in the COVID-19 event was consistent with the homogeneous Poisson process in time distribution. In terms of factors influencing Internet users’ risk perception, economic, education, health level, and degree of information disclosure had significant effects. The regression results in Table 6 also show that all five linear regression models of Internet users’ risk perceptions were statistically significant. In terms of influencing factors:

1. *The level of economic development had a significant effect on the risk perception of Internet users*

The gross regional product per capita in the Internet users’ region had a statistically significant effect on the level of risk perception of Internet users during the evolution of COVID-19 events. Internet users in regions with higher economic levels had a greater demand for risk information, and their resources for understanding and defending against risk events were greater, thus increasing their information search, which is consistent with hypothesis 5.

(2) *The education level of Internet users had a significant effect on the risk perception of Internet users*

The number of years of education per Internet user had a statistically significant effect on the level of Internet users’ risk perception during the evolution of COVID-19 events. In general, the higher the schooling age, the higher the risk perception level, which is consistent with hypothesis 3.

(3) *The health level of Internet users had a significant effect on the risk perception of Internet users*

The annual population mortality rate, which reflects the health level of local residents, had a strong influence on the level of risk perception of Internet users in the first 4 weeks after the COVID-19 outbreak. The lower the population mortality rate in the reflection of health level, the more concerned the Internet users were about being infected by COVID-19, and the higher their risk perception level. Conversely, the higher the mortality rate, the lower the health level of the local region, and the lower the risk perception level of the Internet users, verifying hypothesis 4.

(4) *The degree of government information disclosure had a significant impact on the risk perception of Internet users*

The rate of decrease in the overall risk perception of Internet users reflected, to a certain extent, the effect of the government control of the spread of the avian influenza epidemic, enhancing information disclosure, and channeling public opinion on the event so as to dispel the anxiety and panic of Internet users. The Chinese government has adopted several information disclosure methods in response to risk events: the National Health Commission releases authoritative information about the epidemic daily, and a joint Chinese Internet disinformation platform has been established. Table 6 shows that the degree of government information disclosure had a significant impact on the risk perception of Internet users. As the search density of the COVID-19 event increased, a higher degree of online information disclosure was reflected. The degree of Internet users’ ignorance of the event decreased faster at this juncture of time and the risk perception increased, which was consistent with hypothesis 6.

(5) *Internet users’ gender and age did not have a statistically significant effect on risk perception*

Although judging from the sign of regression coefficients, the regression coefficients of Internet users’ gender and age structure variables were both positive, reflecting that male Internet users have higher risk perception, which verifies hypothesis 1. The higher the proportion of Internet users aged 15–64 in the population age structure, the higher their risk perception, which verifies hypothesis 2; however, the regression results still show that Internet users’ gender and age did not have a statistically significant effect on risk perception.

### Analysis of the temporal evolution of internet users’ risk perceptions

#### Periodic characteristics of internet users’ risk perceptions

Furthermore, we analyzed the spatial and temporal characteristics of the changes in risk perception of Internet users from the above statistical results. From the temporal dimension, considering that the risk perception of Internet users changes daily after the CC, we found that the risk perception index of Internet users in each 4 weeks of the COVID-19 event basically experienced a process of “rapid outbreak - rapid decline - small rebound - steady decline,” (see Fig. 2) showing the characteristics of periodic variations. From Fig. 1, after the first week, the risk perception of Internet users basically showed a monotonic decline, and by the end of the 8th week, the risk perception of Internet users stabilized at a relatively low level, with a complete cycle of about 56 days (8 weeks). The week after the outbreak (7 days) is the key “time window” for risk perception. Figure 1 shows that the risk perception of online netizens and the evolution of the offline COVID-19 epidemic show a largely synchronized trend, and Fig. 1 clearly reflects that the peak of online public opinion preceded the peak of offline epidemic, which also indicates that there was insufficient information disclosure in the early stage of the COVID-19 outbreak and that Internet users had strong doubts, panic, or overreaction of public opinion.

#### Stability trends of internet users’ risk perception

Figures 1 and 2 show that the outbreak of COVID-19 in China at the end of 2019, and overall, the risk perception of COVID-19 among Internet users was irregularly and pulsating distributed. For most of the time, the level of risk perception of COVID-19 among Internet users remained at a high level. However, when Wuhan took the CC measure on January 23, 2020, the Internet users’ risk perception index rapidly jumped to the highest peak in the first week afterwards, and the level of risk perception was much higher than that in other weeks, both in terms of peak information search volume and subsequent development. The peak of the 1st week after the Wuhan CC was more than 30 times of the peak of the 8th week. During the period from the 1st week to the 4th week after the CC in Wuhan, the λ value of each week was very high, among which the 1st week reached 33.7%, reflecting the rapid growth of Internet users’ demand for COVID-19 information and the explosive evolution of Internet users’ emotions. However, after the government adopted an effective prevention and control system and improved the level of information disclosure, the λ value dropped rapidly from 1 month after the Wuhan CC to 0.169 in week 5, about 50% of that in week 1, and to 3.4% in week 8, about one-tenth of that in week 1. This indicates that the overall risk perception of Internet users showed a balanced downward trend (see Table 5). This also indicates that, after the Wuhan CC event, Chinese Internet users were generally adapting to the possible impact of the COVID-19 risk event, and their psychological tolerance had been significantly enhanced as the Chinese prevention and control model continued to play a positive role.

### Analysis of spatial distribution of internet users’ risk perceptions

#### Spatial distribution uniformity characteristics of internet users’ risk perceptions

Table 2 shows that in all five weekly “time windows” (i.e., January 23, week 1, week 2, week 5 and week 8) of the COVID-19 event in 31 provinces, except for the day of the CC, the average value of Internet users’ risk perception of Internet search volume density in the following 8 weeks did not exceed that in the first week (1.31) and continued to decline. The arithmetic mean of the standard deviation of the mean value of Internet search volume density was controlled within 0.38. This indicates that the risk perception of COVID-19 by Internet users in each province is balanced in terms of geographical distribution and is in a basically stable state.

#### Internet users’ risk perception is strongly correlated with the spatial distribution of urban mega-scale and geographic location

Tables 2 and 3 show that Beijing and Shanghai were always in the risk perception zone of level I and II, indicating that the risk perception degree of Internet users in megacities is generally higher. Generally speaking, the degree of Internet users’ risk perception is lower in areas with remote natural geographical locations. From the geographical distribution of risk perception Internet search volume density values of Internet users in each province, including Tibet, the province (region) with outliers excluded from the table, Xinjiang, Yunnan, and Guangxi were always in the lowest level of risk perception of Internet users.

#### Internet users’ risk perceptions generally show the characteristics of the spatial aggregation effect

Based on the IP analysis of Internet search information, Table 3 shows that the spatial aggregation effect of Internet users’ risk perception is obvious, and in more than 80% of provinces, the level of Internet users’ risk perception was clustered in the level III risk perception zone. The spatial aggregation effect of Internet users’ risk perception is also reflected in the fact that certain megacities and provincial capitals are extremely prone to become high-level risk perception areas, such as Beijing, Shanghai, Hangzhou, other cities, and Wuhan, which is not discussed in this paper because of its particularly huge value.

#### Internet users’ risk perceptions and epidemic prevention and control effects in different places show spatial synergy characteristics

Table 3 shows that the risk perception level areas of Internet users are not consistent with the epidemic level of each province online. In the first and second weeks after the CC, the weekly new confirmed cases in Zhejiang, Anhui, Hunan, Heilongjiang, Henan, Guangdong, Shandong, Chongqing, and Jiangsu provinces (cities) were above 200 cases, far more than other provinces in the same level III risk perception area of Internet users. However, the Internet search density values of these provinces (cities) with more new confirmed cases per week were relatively stable. This is closely related to the effect of strengthening epidemic prevention and control and public opinion management online and offline in these provinces, reflecting the spatial synergy between Internet users’ risk perception and the effect of epidemic prevention and control in each place.

### Analysis of the impact of space-time interaction of internet users’ risk perception

#### Hot topics of internet search tend to stabilize

Table 4 shows that in the ranking results of the hot topics that Internet users were concerned about regarding COVID-19, most of the 28 hot topics that entered the weekly search volume of more than 500 million items was the 8th week, and five items entered the top 8% of hot topics in the 8th week, indicating that the hot topics gradually tend to be concentrated. There were three hot topics in week 1, 3, 6, and 7, and four hot topics in week 4, indicating that most of the time, the hot topics of Internet users are relatively stable, and the level of risk perception of Internet users does not have a large space-time mutation due to the influence of space-time interaction.

#### The structure of internet users’ information demand tends to be rational

Table 4 also shows that in the five important “time windows” of risk perception of COVID-19 Internet users, the change of Internet users’ demand for three types of information, namely, the latest COVID-19 news, COVID-19 evolution prediction and prevention and control, and COVID-19 transmission trend, becomes more rational with the spatial and temporal evolution. Table 4 shows that in the 8 weeks after the CC order was issued, Internet users in various provinces paid attention to category 1 of hot topics of public opinion, accounting for 53.6% of the total number of the top 8% hot topics (28), reflecting a high degree of concern about the COVID-19 infection and the effectiveness of government control. Category 2 public opinion hot topics accounted for 28.6% of the total number of the top 8% hot topics, reflecting the rational concern of the Internet users about the development trend of the epidemic as judged by experts. Category 3 public opinion hot topics accounted for 17.9% of the total number of the top 8% hot topics, reflecting the concern of the Internet users about the transmission and importation from abroad. Combined with the comprehensive analysis of the time window of these hot topics in Table 4, it is not difficult to find that the Internet users’ concern about the government’s ability to control COVID-19 and the future trend of COVID-19, especially the potential threats such as the risk of overseas transmission, far exceeds the impact of rumors and excessive emotions at the beginning of the outbreak. This also reflects another aspect of the increasing rationale of Chinese Internet users’ information demand structure in the face of COVID-19 major public health events.

## Discussion

The space and time distribution law of Internet users’ risk perceptions in major public health events of COVID-19 as the research object was applied to Internet search volume of Internet users in major public health events as a proxy variable for Internet users’ risk perceptions to analyze the evolution characteristics of Internet users’ risk perception variables in time and space dimensions and their influencing factors. Considering that Internet users’ Internet search data is the risk perception data of each week for the specific event of COVID-19 and has non-linear characteristics, we adopted five linear regression models according to different time periods to analyze the space and time distribution of Internet users’ risk perception. The results showed that economic, educational, and health levels and the degree of information disclosure had significant effects on the risk perceptions of Internet users. The evolution of risk perceptions of Internet users during the COVID-19 event conformed to the exponential distribution law in time with the characteristics of periodicity and stability and had the basic characteristics of uniformity of distribution in space, a strong correlation between city scale and geographic location, spatial aggregation, and spatial synergy with the effect of epidemic prevention and control. The results of the space-time interaction of Internet users’ risk perceptions show that Chinese Internet users’ Internet search for hot topics was more stable and the structure of their information demands was more rational in the face of the major public health event of COVID-19.

## Conclusions

These findings have important practical significance. First, the Internet search cycle of the COVID-19 event is synchronized with the evolution cycle of the epidemic itself, and in this process, the physical risk of Internet users is always at the top of the risk structure, focusing on the strong concern shown by Internet users about the government’s ability to control COVID-19 and the future trend of COVID-19. Therefore, it is advisable for the government to expand the openness of the Internet, activate online information sources, combat the spread of rumors, strengthen the control and governance of major public health events themselves, and do a good job of authoritative information dissemination. Second, it is best to establish a data-driven, risk-aware intelligent system for Internet users. Based on the exponential function distribution and periodic characteristics of the evolution of Internet users’ risk perception in the COVID-19 event, the government should use massive Internet search data to research and develop a big data-driven early warning system for Internet users’ risk perception, so as to understand the state of Internet users’ risk perception in a timely manner and guide their risk decision-making behavior rationally. Third, the government should seize the risk control focus of key time nodes, key regional locations, and key information contents of online communication, actively adjust the information content supply, effectively control the rebound of Internet users’ risk perception, and guide people to actively face and overcome the potential risks and threats brought about by COVID-19.

## Data Availability

The datasets generated and/or analyzed during the current study are available in the China Statistical Yearbook repository, (http://www.stats.gov.cn/tjsj/ndsj/2019/indexch.htm).

## Abbreviations

CC: City closing
PRC: People’s Republic of China
COVID-19: Coronavirus disease 2019
